# Population genetic analysis of the liver fluke *Fasciola hepatica* in German dairy cattle reveals high genetic diversity and associations with fluke size

**DOI:** 10.1186/s13071-025-06701-6

**Published:** 2025-02-13

**Authors:** Anna Sophie Hecker, Marie-Kristin Raulf, Sven König, Katharina May, Christina Strube

**Affiliations:** 1 Institute for Parasitology, Centre for Infection Medicine, University of Veterinary Medicine Hannover, Hannover, Germany; 2 Institute of Animal Breeding and Genetics, Justus-Liebig-University Gießen, Gießen, Germany

**Keywords:** *cox1*, *nad1*, Haplotype, Genotype, Population analysis, Genetic diversity, Clones, Morphometry, Fluke size

## Abstract

**Background:**

The liver fluke *Fasciola hepatica* is one of the most important endoparasites in domestic ruminants worldwide and can cause considerable economic losses. This study presents the first population genetic analysis of *F. hepatica* in Germany and aims at providing new insights into genetic diversity and population structure.

**Methods:**

A total of 774 liver flukes, collected from 60 cows of 17 herds and 13 cows of unknown herd origin, were subjected to comparative analysis of two mitochondrial genes (*cox1* and *nad1*), one nuclear region (internal transcribed spacer (ITS)-1) and eight nuclear microsatellite markers. In addition, individual fluke measurements allowed comparison of morphometric differences between genotypes.

**Results:**

The nuclear ITS-1 region showed minimal variability, with 772 of 774 flukes having identical sequences, while the mitochondrial sequences revealed a high genetic diversity, with 119 distinct haplotypes, a mean haplotype diversity (Hd) of 0.81 and a mean nucleotide diversity (*π*) of 0.0041. Mitochondrial phylogenetic analysis identified two clusters with no clear association with the host or farm of origin. In the microsatellite analysis, all eight loci were highly polymorphic, with a mean allele frequency of 19.0 and a mean genotype frequency of 73.5 per locus. A total of 500 unique multilocus genotypes (MLGs) were found across all fluke samples, indicating that 68.5% of all genotypes were unique. A mean expected heterozygosity of 0.71 suggested a high potential for adaptability and the number of migrants (Nm = 3.5) indicated high gene flow between farms. Population structure analysis based on microsatellite data revealed that flukes from two farms differed genetically from the others. Linear mixed model results revealed that fluke length differed significantly between the two mitochondrial clusters, although it should be noted that fluke age could not be considered in the analyses.

**Conclusions:**

*Fasciola hepatica* in German dairy farms showed high genetic diversity and gene flow. The differences in population structure identified by mitochondrial sequences compared with microsatellite loci highlight the benefits of analysing genetic markers of different origins. This is the first study to correlate fluke morphometry measurements with genetic markers, indicating that the identified markers can influence fluke size.

**Graphical Abstract:**

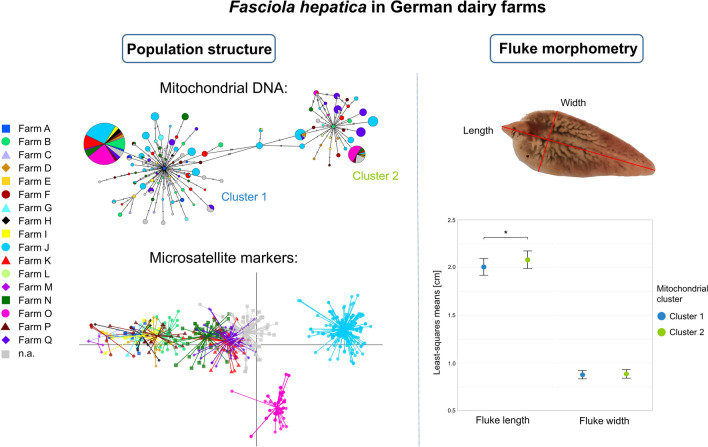

**Supplementary Information:**

The online version contains supplementary material available at 10.1186/s13071-025-06701-6.

## Background

The trematode *Fasciola hepatica*, also known as the common liver fluke, is a globally distributed endoparasite primarily found in ruminants. Cattle infected with *F. hepatica* may exhibit chronic symptoms such as diarrhoea, anaemia or weight loss [1]. In addition, infections can result in reduced milk yield and fertility, causing financial losses for dairy farmers [2–5]. In Germany, recent studies on farm-level have reported liver fluke infections in 10% of cattle farms based on copromicroscopic examinations [6] and a seroprevalence of 15% [7]; this is comparatively lower than in other European countries, where seroprevalences of up to 93% have been reported [8–10].

The life cycle of *F. hepatica* is complex, involving mud snails as intermediate hosts and a wide range of mammalian definitive host species [11]. With its faeces, the definitive host excretes eggs from which miracidia hatch. Within the intermediate snail host, e.g. *Galba truncatula*, the miracidia develop into sporocysts, rediae and cercariae and undergo clonal expansion [12]. The cercariae are released from the snail, encyst into metacercariae on pasture and are ingested by their definitive host, e.g. domestic and wild ruminants. Newly excysted juvenile flukes migrate through the intestinal mucosa, peritoneal cavity and liver parenchyma until they reach the bile ducts, where they mature into adults [11]. As a hermaphrodite, *F. hepatica* is capable of both self-fertilisation and cross-fertilisation in the definitive host, although previous studies have shown that self-fertilisation rarely occurs [13–15].

The complex life cycle of *F. hepatica*, its wide host range and global distribution provide an interesting framework for population genetic analyses. Knowledge of genetic variation and diversity as well as population structure aids in understanding the distribution of parasites, their potential to adapt to new environmental conditions and how new adaptive traits, such as drug resistance, can spread through a population [16]. Most population genetic analyses of *F. hepatica* are based on sequencing of mitochondrial gene regions. Mitochondrial DNA (mtDNA) is haploid, uniparentally inherited, non-recombining and contains numerous protein-coding genes [17]. It is frequently used in the field of population genetics, primarily owing to its high mutation rate in comparison with nuclear DNA, such as the internal transcribed spacer 1 or 2 (ITS-1/ITS-2) [18, 19]. Among the most extensively studied mitochondrial markers are the cytochrome C oxidase subunit 1 (*cox1*) and the nicotinamide dehydrogenase subunit 1 (*nad1*) genes. Previous studies using these markers have shown that mitochondrial genetic diversity varies considerably between geographic regions. Reported *F. hepatica* haplotype diversity (Hd), which describes the chance of observing two different haplotypes when randomly sampling two specimens from a population, ranged from 0.43 (*cox1*) and 0.31 (*nad1*) in Algeria [20] to 0.93 (*cox1*) and 0.93 (*nad1*) in Spain [21]. By retrospectively analysing 604 *F. hepatica cox1* and *nad1* gene sequences from around the world, Alvi et al. [22] found that some geographic regions have specific variants. Accordingly, region-specific sequence variations were found in Iran [23] and between European and African *F. hepatica* [24]. Studies comparing *F. hepatica* genetics within a particular geographic region discovered genetic differences between fluke populations from different locations in Brazil [25], but no geographical structuring was found in flukes from Eastern Europe, Western Asia [26], China [27] or Argentina [28].

Despite being a popular tool in population genetics, the use of mtDNA alone has attracted some criticism, as mtDNA is considered a single locus and may be strongly influenced by genetic selection [29, 30]. Alternatively, nuclear microsatellite markers have proven to be a useful tool for detecting genetic variation. Microsatellite markers are maternally and paternally inherited, co-dominant and highly polymorphic, rendering them suitable for resolving population structures [31, 32]. Hurtrez-Boussès et al. [33] described the use of five polymorphic microsatellite markers to investigate the population structure for *F. hepatica* in Bolivia. These markers have also been used in studies in Egypt [34], Spain [35, 36] and Cuba [37]. Cwiklinski et al. [16] developed a novel microsatellite panel of 15 highly polymorphic loci, which provided consistent results and exhibited a high degree of polymorphism for *F. hepatica* found in British cattle. This panel has also been successfully implemented in further studies, showing a lack of population structure in flukes from Cuba [37] and the UK [14], but some geographic structuring in Spain [35] and Argentina [15].

The opposing results of population genetic analysis in different countries highlight the need for region-specific studies. This is the first study on population genetics of *F. hepatica* in Germany and aims to provide new insights into genetic diversity and population structure of liver flukes in German dairy farms. One nuclear region (ITS-1), two mitochondrial genes (*cox1* and *nad1*) and eight nuclear microsatellite markers [16] were analysed to obtain comprehensive data by comparative analysis of nuclear and mitochondrial markers [31, 32, 38, 39]. Furthermore, genotypic data were associated with fluke morphometry to determine whether the identified genetic markers had an effect on fluke size.

## Methods

### Liver fluke collection and phenotyping

Infected livers (*n* = 73) of dairy cows were collected at different abattoirs in northern and central Germany (located in the federal states of Lower Saxony, Schleswig–Holstein and Hesse) between June 2021 and January 2023. While 52/73 livers originated from 14 farms with known locations (labelled farms A to N, Additional file 1: Supplementary Fig. 1), and 8/73 livers could be assigned to three farms with unknown location (farms O, P and Q), 13/73 livers could not be traced to any farm and were therefore labelled only according to their host ID (×1 to ×13). All adult flukes visible in the bile ducts or gall bladder of the infected livers were collected (*n* = 774). Fully (*n* = 562) and partially (*n* = 60) intact liver flukes were measured under a stereomicroscope (ZEISS SteREO Discovery.V8, Carl Zeiss, Oberkochen, Germany) immediately after their extraction. Some flukes (*n* = 152) could not be measured because they were incomplete or decomposed. Measurements included body length (*n* = 577), body width (*n* = 621), the ratio of body length to body width (*n* = 576) and the distance between the ventral sucker and the posterior end of the fluke (*n* = 562). After phenotyping, flukes were frozen at −20 °C until DNA extraction. Statistical tests were performed in R version 4.2.3 [40]. To test whether the number of flukes present in a liver influenced fluke size, Spearman’s rank correlation (*ρ*) was calculated. Differences in fluke length and width between farms and mitochondrial clusters were assessed using linear mixed models with the lme4 package version 1.1–36 in R [41]. Farm L was excluded from the analysis because only one fluke was sampled from this farm. Model selection was done by comparing the Akaike information criterion (AIC) and residual plots. The final models included farm and mitochondrial cluster as fixed effects and fluke count of the liver, sampling month and host as random effects. Interactions between farm and mitochondrial cluster were tested but were not significant; therefore, the interaction was removed from the models. The final models were significantly different from a null model containing only the random effects (*P* = 0.003 for the fluke length model and *P* = 0.001 for the fluke width model). Type III Wald chi-squared tests were performed to assess the significance of the fixed effects. The emmeans package version 1.10.6 [42] was used to calculate least-squares means, i.e. means adjusted for the other variables in the models, for the fixed effects ‘farm’ and ‘mitochondrial cluster’. Pairwise comparisons with a Tukey correction were conducted between least-squares means of the mitochondrial clusters and between least-squares means of all farms.

### Fluke DNA isolation and ITS-1, *cox1* and *nad1* sequencing

Genomic DNA was isolated from individual flukes using the NucleoSpin 96 Tissue Kit (Macherey Nagel, Düren, Germany) according to the manufacturer’s instructions. To avoid contamination by eggs or sperm, 10 mg of the anterior end of the fluke was used [14]. For the subsequent PCR runs, primer pairs for ITS-1 (ITS1-F and ITS1-R), *cox1* (Ita8 and Ita9) and *nad1* (Ita10 and Ita2) sequences were adopted from Itagaki et al. [43] and are displayed in Table 1. Each 25 µl reaction set-up contained 0.125 µl Dream Taq polymerase (5 U/µl) (Thermo Fisher Scientific, Waltham, Massachusetts, USA), 2.5 µl DreamTaq Buffer (10x) (Thermo Fisher Scientific, Waltham, Massachusetts, USA), 0.5 µl dNTPs (10 mM) (Carl Roth, Karlsruhe, Germany), 1.0 µl forward primer (10 µM) and 1.0 µl reverse primer (10 µM). For amplification of the ITS-1 sequence, 3.0 µl genomic DNA was added as template, while 1.0 µl genomic DNA was added for amplification of the *cox1* and *nad1* gene. Reaction cycles consisted of an initial denaturation at 95 °C for 3 min, followed by 35 cycles of denaturation at 95 °C for 30 s, primer annealing at 55 °C (ITS-1) or 53 °C (*cox1* and *nad1*) for 30 s, extension at 72 °C for 60 s and a final extension at 72 °C for 10 min. Successful PCR amplification was verified by gel electrophoresis using 1.5% agarose gels supplemented with GelRed (dilution 1:10,000, Merck, Darmstadt, Germany). Expected amplicon sizes were 680 bp for ITS-1, 493 bp for *cox1* and 660 bp for *nad1*. The PCR products were then subjected to custom Sanger sequencing (Microsynth Seqlab GmbH, Göttingen, Germany). Upon unsuccessful sequencing of the *nad1* gene, PCR was repeated with the newly designed primer pair Nad1 (Table 1, designed using NCBI Primer-BLAST [44]), which spanned a 936 bp sequence. Sequences were deposited in NCBI GenBank under accession numbers PQ742167-PQ742169 (ITS-1), PQ742177-PQ742950 (*cox1*) and PQ759109-PQ759882 (*nad1*).

**Table 1:** Primer pairs used for the amplification of *F. hepatica* ITS-1, *cox1*, *nad1* and microsatellite loci

Gene/locus	Primer sequence (5′-3′)	Fluorescent dye	References
ITS-1	ITS1-F: 5′-TTGCGCTGATTACGTCCCTG-3′	–	Itagaki et al. [43]
	ITS1-R: 5′-TTGGCTGCGCTCTTCATCGAC-3′		
*cox1*	Ita8: 5′-ACGTTGGATCATAAGCGTGT-3′	–	Itagaki et al. [43]
	Ita9: 5′-CCTCATCCAACATAACCTCT-3′		
*nad1*	Ita10: 5′-AAGGATGTTGCTTTGTCGTGG-3′	–	Itagaki et al. [43]
	Ita2: 5′-GGAGTACGGTTACATTCACA-3′		
*nad1*^a^	Nad1_for: 5′-TGTTGCAGAGGTTTGCGGA-3′	–	This study
	Nad1_rev: 5′-CCCCCAGAAATACCGACGAA-3′		
Fh_2	F: 5′-TGAGAAACTGATTCACCGACTG-3′	ATTO565	Cwiklinski et al. [78]
	R: 5′-GAGCTTGTGCTCTCGGAACTA-3′		
Fh_5	F: 5′-CATCACCACTGTCTTCGATCA-3′	FAM	Cwiklinski et al. [78]
	R: 5′-CGAAGCATTGATAAGATTTCCA-3′		
Fh_6	F: 5′-ACGTCCGTCCGTTAAGTGAG-3′	ATTO550	Cwiklinski et al. [78]
	R: 5′-TTTGAGGTCGACATCCTTCA-3′		
Fh_10	F: 5′-TTTAGTCGCGGAGCTACCAT-3′	ATTO532	Cwiklinski et al. [78]
	R: 5′-CCACTTTCGTCATGCACATT-3′		
Fh_11	F: 5′-TAAACCGTTGCTTCACGTTG-3′	ATTO550	Cwiklinski et al. [78]
	R: 5′-CAAAGTGTTTGGCGAGCTG-3′		
Fh_12	F: 5′-CCACGAGAAGTGGAATTCGT-3′	ATTO532	Cwiklinski et al. [78]
	R: 5′-GTAGGTCCACTCCCTGTCCA-3′		
Fh_13	F: 5′-GAAACTGTCCCGAAAACGAG-3′	FAM	Cwiklinski et al. [78]
	R: 5′-GCGTGCAACATAGGTGAAAA-3′		
Fh_15	F: 5′-AATGCGGAAAAGAGCGATTA-3′	ATTO565	Cwiklinski et al. [78]
	R: 5′-GAAATTGGGAGCAACTGCAT-3′		

^a^ The Nad1 primer pair was used when the Ita10/Ita2 primer pair did not result in successful amplification or sequencing

### Analysis of ITS-1, *cox1* and *nad1* sequences

Sequences were trimmed to equal length, i.e. 568 bp for ITS-1, 410 bp for *cox1* and 582 bp for *nad1*, and aligned using Clone Manager 9 (Scientific & Educational Software, Cary, NC, USA) and UGENE [45]. *Cox1* and *nad1* reference sequences from different countries were retrieved from NCBI GenBank. The number of polymorphic sites, synonymous and non-synonymous substitutions and the number of haplotypes were calculated using the DNA Sequence Polymorphism (dnaSP) software version 6 [46]. The same program was used to calculate nucleotide diversity (*π*), defined as the mean number of nucleotide differences per site, and Hd, which describes the probability that two randomly selected haplotypes are distinct. Hd ranges from 0 to 1, with a value of 1 signifying the highest possible diversity. *Cox1* and *nad1* sequences from each fluke were merged using SequenceMatrix [47], resulting in sequences of 992 bp. To test for neutral evolution of a population, neutrality indices (Tajima’s D, Fu’s Fs and Fu and Li’s D and F tests) were calculated [48] by dnaSP software version 6 [46]. Maximum-likelihood trees were constructed with IQ-TREE [49] using ModelFinder to find the best fitting model [50] and applying ultrafast bootstrap approximation (1,000 bootstraps) [51]. Trees were then formatted using iTOL [52]. A minimum spanning network of haplotypes from the merged *cox1* and *nad1* sequences, grouped by their farm of origin, was created using PopART [53, 54]. To calculate the influence of host and farm on genetic variation, an analysis of molecular variance (AMOVA) with 10,000 permutations of flukes grouped by host and farm (if information on the farm of origin was available) was performed using Arlequin version 3.5.2.2 [55]. As part of the AMOVA analysis, the fixation index F_ST_, which serves as a measure of population differentiation, was calculated. An F_ST_ of 0 indicates the complete absence of population structure, whereas an F_ST_ of 1 signifies completely distinct subpopulations.

### Microsatellite multiplex PCR

The eight best performing loci of a microsatellite panel developed by Cwiklinski et al. [16] were selected (Fh_2, Fh_5, Fh_6, Fh_10, Fh_11, Fh_12, Fh_13 and Fh_15). Multiplex PCR and fragment size detection were custom performed by Microsynth (Microsynth AG, Balgach, Switzerland). Briefly, the eight microsatellite markers were analysed in two multiplex reactions (multiplex assay one: Fh_5, Fh_11, Fh_12 and Fh_15 and multiplex assay two: Fh_2, Fh_6, Fh_10 and Fh_13). Loci were amplified in a reaction volume of 10 µl with 2.0 µl Hot FirePol Multiplex Mix (5×) (Solis BioDyne, Tartu, Estonia), 1.5 µl forward primer (2 µM), 1.5 µl reverse primer (2 µM), 3.0 µl ddH_2_0 and 2.0 µl of genomic DNA (1–5 ng/µl). Primer sequences and fluorescent dyes are shown in Table 1. Cycling conditions consisted of an extended denaturation step at 95 °C for 12 min, followed by 35 cycles at 95 °C for 20 s, 60 °C for 50 s, and 72 °C for 120 s and a final extension at 72 °C for 5 min. Fragment size was determined using GeneScan LIZ500 Dye Size Standard (Applied Biosystems, Thermo Fisher Scientific, Waltham, Massachusetts, USA), and fragment analysis was performed on the Applied Biosystems 3730XL Series Genetic Analyzer (Thermo Fisher Scientific, Waltham, Massachusetts, USA) with 10 s injection time, 1.6 kV injection voltage, 2100 s run time, 15 kV run voltage, 50 cm capillary length, POP7 polymer and Dye Set G5 filter.

### Analysis of microsatellite data

Allele and genotype frequencies for each locus were determined using the genepop package version 1.2.2 [56] in R version 4.2.3 [40]. Observed and expected heterozygosity as well as null allele frequency to account for alleles that did not amplify in PCR, e.g. by mutations at the primer site, were estimated for each locus using CERVUS version 3.0.7 [57]. For each individual fluke, allele lengths from all loci were combined into multilocus genotypes (MLGs). For each farm, the number of distinct MLGs and the expected heterozygosity (Nei’s unbiased gene diversity) [58] were calculated using the poppr package version 2.9.6 for R [59]. Genotypic richness (*R*) on each farm was calculated as follows: *R* = (number of MLGs-1)/(number of fluke samples-1) [14]. A value of 0 signifies the existence of only a single MLG within a given farm (all flukes are clones) and 1 indicates that each fluke from that farm has a unique MLG (no clones). For further analysis, MLGs were reduced to one instance per host using poppr version 2.9.6 [59], resulting in a clone-corrected dataset containing 554 MLGs.

Deviations from Hardy–Weinberg equilibrium (HWE, describing an ideal population where no evolutionary influences exist) were estimated by performing a probability test using the Markov chain method (10,000 dememorization, 250 batches, and 5000 iterations) and by calculating F_IS_ values [60] in genepop version 1.2.2 [56]. The inbreeding coefficient F_IS_, which ranges from −1 to 1, is used to describe the agreement between observed and expected genotypic frequencies, thus measuring heterozygote excess (negative values) or deficiencies (positive values) [60]. The testing of each locus for deviations from HWE was also performed separately for each farm (486 MLGs from 17 farms) or host ID, if the farm of origin was unknown (68 MLGs from 13 hosts), to further investigate observed deviations.

Pairs of loci were checked for linkage disequilibrium (LD), i.e. the probability of two alleles occurring together at different gene loci, across all samples using poppr [59]. For this purpose, the index of association (*I*_*A*_) [61] and the standardised index of association (*r*_*d*_), which is independent of sample size [62], were calculated with 1000 permutations. LD was also assessed separately for each farm (or host ID if the farm of origin was unknown) on the basis of a likelihood ratio test [63] with 20,000 permutations, using Arlequin version 3.5.2.2 [55]. To correct for multiple testing, *P*-values for LD were adjusted using the Bonferroni correction [14, 64].

The rate of self-fertilisation (*s*) was estimated from the inbreeding coefficient F_IS_, which was calculated across all loci in genepop version 1.2.2 [56], according to the following equation: *s* = 2*F_IS_/(1 + F_IS_) [65, 66]. To assess the influence of host and farm on genetic variation and to calculate the fixation index F_ST_, an AMOVA with 10,000 permutations was performed using Arlequin version 3.5.2.2 [55] on the whole and the clone-corrected dataset. The frequency of private (unique) alleles can be used as an estimator of individuals exchanged between populations, i.e. the number of migrants, and therefore, as a measure of gene flow [67]. The number of migrants (Nm) between farms corrected for sample size (mean number of flukes per farm) was determined after grouping the fluke samples by farm or, when the farm of origin was unknown, by host ID using genepop version 1.2.2 [56].

Two different methods were used to assess and visualise population structure, i.e. the difference in allele frequencies between subpopulations. A Bayesian clustering approach was performed without prior information on the origin of the sample using STRUCTURE 2.3.4 (200,000 Burn-in period, 100,000 Markov Chain Monte Carlo repeats and *K* = 1–30, 20 iterations each) [68] to create admixture heritage models for multiple possible numbers of populations (*K*). The pophelper package version 2.3.1 for R [69] was used to analyse the results, applying the Evanno method [70] to assess the most likely *K*. As a multivariate approach, a discriminant analysis of principal components (DAPC) was conducted using adegenet version 2.0.0 for R [71] and visualised in 3D using the plotly package version 4.9.3 [72]. For this analysis, the flukes were grouped according to their farm of origin, while those with unknown origin were grouped together. To mitigate the bias of unequal sample sizes between farms, a population size-based weighting strategy was applied using the vegan package version 2.6–8 for R [73]. For each individual, a weight was assigned that inversely correlated with the population size, ensuring that smaller populations were given greater weight in the analysis. The weights were adjusted using a power transformation with an exponent of 0.25 to avoid distortion owing to overly aggressive corrections. After visual inspection of the DAPC plot, one individual (the only fluke collected from farm L) was identified as an outlier, which appeared to distort the overall population structure. As a result, this individual was excluded from the DAPC.

## Results

### Liver fluke collection

The number of flukes in the infected livers (*n* = 73) ranged from 1 to 91 flukes per liver, with a mean of 10.6 (95% confidence intervals [CI] 7.1–14.1) and a median of 5.0 (95% CI 4.0–8.0). A total of 774 adult *F. hepatica* were collected from the bile ducts for genotyping and phenotyping. Livers and liver flukes for which the farm of origin was known (693 *F. hepatica* specimens from 60 livers) are listed in Table 2.

**Table 2:** Farm specific data on genetic diversity derived from merged *cox1* and *nad1 F. hepatica* sequences (992 bp). Only data from flukes with known farm of origin were included (693 liver flukes from 17 farms)

Farm	No. of livers	No. of flukes	Nucleotide diversity (*π*)	No. of haplotypes	Haplotype diversity (Hd)
Farm A	2	12	0.0026	5	0.83
Farm B	8	50	0.0018	11	0.63
Farm C	1	6	0.0040	4	0.80
Farm D	3	14	0.0022	6	0.74
Farm E	1	4	0.0067	3	0.83
Farm F	3	8	0.0005	2	0.54
Farm G	1	11	0.0061	5	0.78
Farm H	2	13	0.0024	3	0.30
Farm I	2	18	0.0048	8	0.70
Farm J	10	233	0.0044	29	0.84
Farm K	9	64	0.0021	13	0.67
Farm L	1	1	n.a.	1	n.a.
Farm M	2	4	0.0000	1	0.00
Farm N	7	48	0.0059	14	0.81
Farm O	3	110	0.0032	4	0.44
Farm P	2	23	0.0047	11	0.91
Farm Q	3	74	0.0024	15	0.90
Mean	3.5	40.8	0.0041	7.9	0.81

n.a.: not applicable

### ITS-1, *cox1* and *nad1* sequences of German *F. hepatica*

All three genetic markers were successfully amplified and sequenced from all liver flukes (*n* = 774). A comparison of the ITS-1 sequences with reference sequences in GenBank confirmed that all 774 parasites were *F. hepatica*. The ITS-1 region showed very little variation between individuals, with 772 out of 774 flukes having identical sequences. The two divergent flukes showed two nucleotide substitutions each, one in the ITS-1 region at the same position but with different base substitutions, and the other in the flanking 5.8 S rDNA region at different positions. Given the absence of intra-species variability, the ITS-1 sequences were excluded from further analysis. The *cox1* and *nad1* sequences exhibited 48 and 69 polymorphic sites, representing 11.7% (48/410) and 11.9% (69/582) of nucleotide positions, respectively. Nucleotide diversity *π* was 0.0047 (*cox1*) and 0.0036 (*nad1*). In the *cox1* sequences, 25.0% of substitutions (12/48) were non-synonymous, i.e. resulting in a changed translated amino acid. The percentage of non-synonymous substitutions in the *nad1* sequences was 40.6% (28/69). The analysis of the *cox1* sequences revealed 53 distinct haplotypes with an Hd of 0.64. From the *nad1* sequences, 78 haplotypes with an Hd of 0.67 were distinguished. Since both genes are of mitochondrial origin and therefore represent one locus [29], the *cox1* and *nad1* sequences from each fluke were fused. An alignment of these merged sequences revealed 11.8% polymorphic sites (117/992), and *π* was 0.0041. A total of 119 distinct haplotypes with an Hd of 0.81 were identified. Among farms, *π* and Hd varied considerably, with *π* ranging from 0.0000 to 0.0067 and Hd ranging from 0.00 to 0.91 (Table 2). All four neutrality tests were uniformly negative, indicating a deviation from the neutral model, in which all mutations are random and no selection occurs (Tajima’s D = −2.2 [*P* < 0.001], Fu’s Fs = −141.1 [*P* < 0.001], Fu and Li’s *D* test = −2.8 [*P* = 0.003] and Fu and Li’s F test = −3.0 [*P* = 0.001]).

**Fig. 1:**
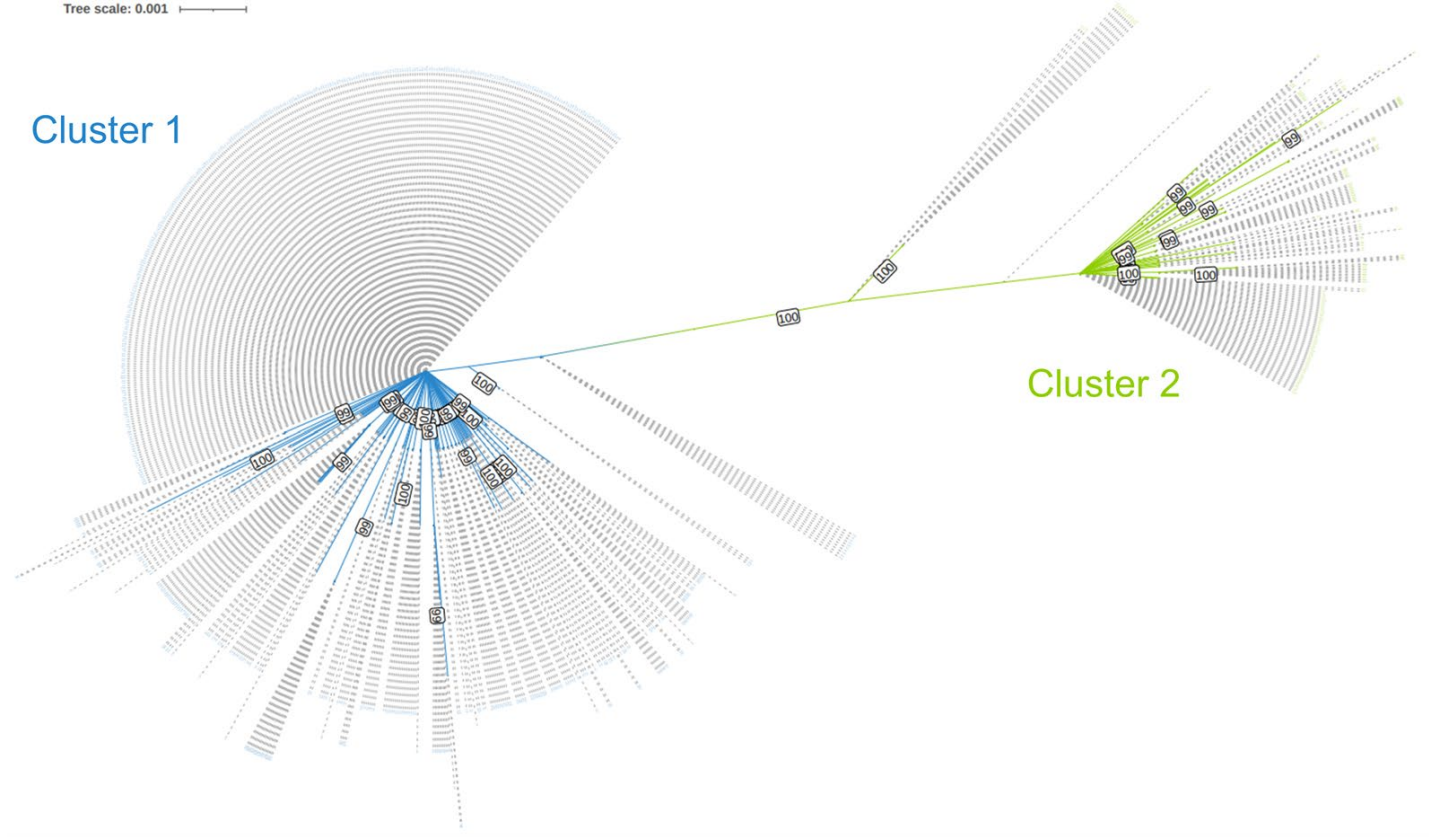
Maximum-likelihood consensus tree constructed from merged *cox1* and *nad1* sequences of German *F. hepatica* (*n *= 774) in IQ-TREE. Bootstrap values ≥ 99 are shown

**Fig. 2:**
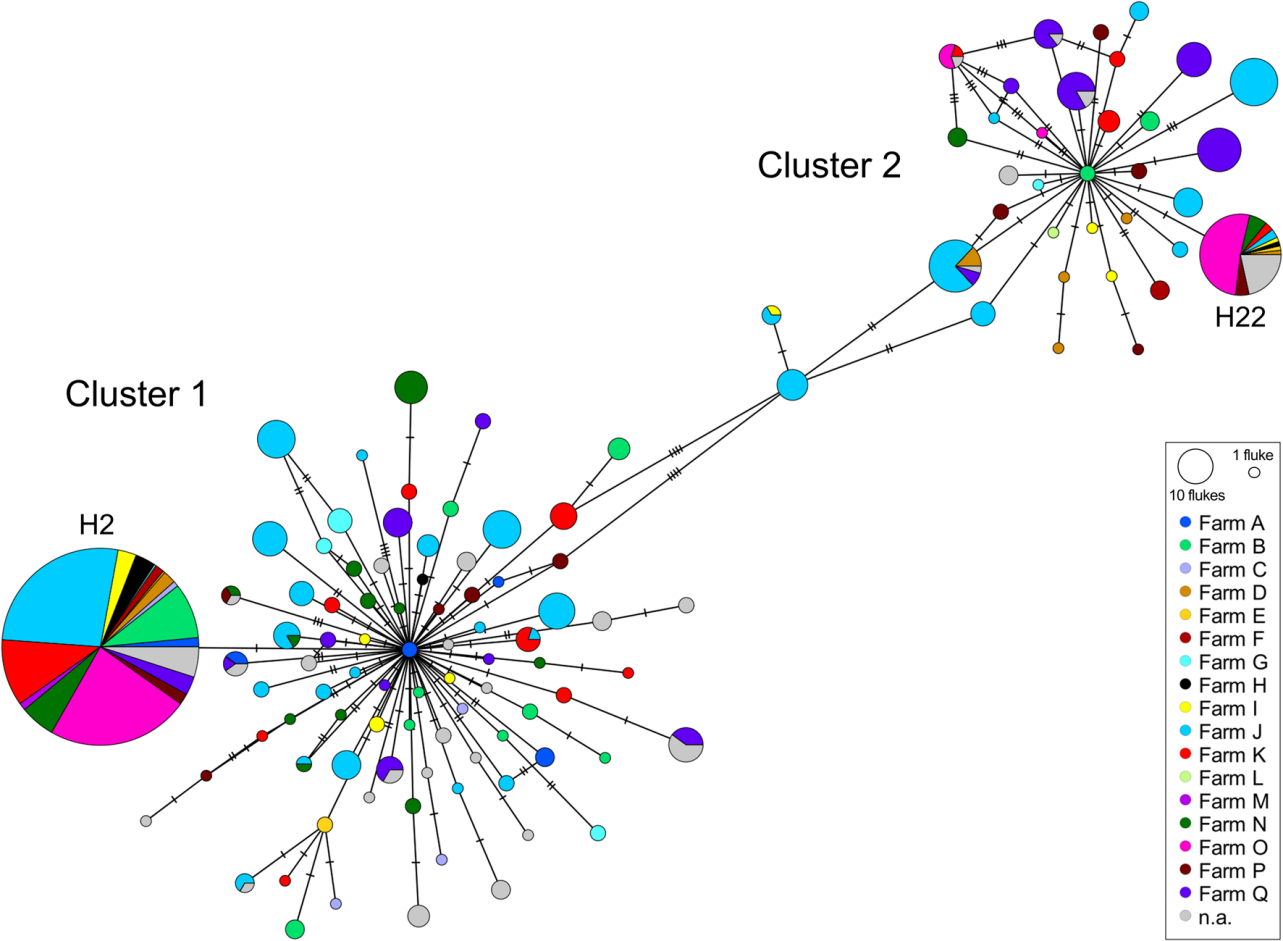
A minimum spanning network constructed from haplotype data of the merged *cox1* and *nad1* sequences of German *F. hepatica* (*n* = 774). Pie charts show the proportion of farms in which each haplotype occurred. The two most common haplotypes (H2 and H22) are labelled. n.a.: not applicable as the farm of origin was unknown

Phylogenetically, the *cox1* and *nad1* sequences were divided into two clusters as depicted in the maximum-likelihood tree (Fig. 1). With 77.9% (603/774) the majority of collected *F. hepatica* were assigned to cluster 1 and 22.1% (171/774) to cluster 2, although 14 of these 171 flukes were slightly separated. A minimum spanning network constructed from the haplotype data of the merged *cox1* and *nad1* sequences showed that the first cluster comprised 84 haplotypes and the second cluster 35 haplotypes (Fig. 2). There was one main haplotype in each cluster: H2 in cluster 1 (42.4% of all flukes, 328/774) and H22 in cluster 2 (7.2% of all flukes, 56/774), which differed by eight nucleotide positions. Most farms (88.2%, 15/17) were represented in both clusters and also around half of hosts (53.4%, 39/73) harboured flukes from both clusters. The AMOVA revealed that the host explained 7.4% (*P* < 0.001) and the farm 10.1% (*P* < 0.001) of the genetic variation, whereas the remaining 82.5% of variation occurred within hosts (Table 3). Overall, F_ST_ was 0.18, which indicates a low degree of structuring by subpopulations. A phylogenetic comparison of the two main haplotypes with other *cox1* and *nad1* sequences from around the world confirmed the substantial phylogenetic distance between the two clusters identified in this study, as the two haplotypes showed a closer relation to sequences from other countries than to each other (Additional file 2: Supplementary Fig. 2).

**Table 3:** Analysis of molecular variance (AMOVA) of the merged *cox1* and *nad1* sequences of *F. hepatica* (*n* = 774). Samples were grouped by host and farm of origin

Source of variation	Sum of squares	Variance components	Percentage variation (%)	*P*-value
Among farms	414.3	0.2	10.1	< 0.001
Among hosts within farms	207.7	0.2	7.4	< 0.001
Within hosts	2486.1	1.7	82.5	< 0.001
Total	3108.1	2.0	100	n.a.

n.a.: not applicable

### Microsatellite data

Microsatellite analysis was successful for at least one locus in 760/774 liver flukes (98.2%) and for all eight loci in 730/774 (94.3%) (Additional file 3: Supplementary Table 1). All eight loci were highly polymorphic, with 11 to 34 alleles per locus (mean: 19.0) and 20 to 150 genotypes per locus (mean: 73.5) (Table 4). Observed heterozygosity varied between 0.45 and 0.88 per locus (mean: 0.73) and expected heterozygosity between 0.50 and 0.90 per locus (mean: 0.76). The estimated null allele frequencies ranged from 0.0% to 5.9% (mean: 2.5%) per locus, indicating a successful amplification and detection of all markers.

**Table 4:** Results of the microsatellite analysis in *F. hepatica* from Germany sorted by locus

Locus	Repeat motif	No. of samples	No. of alleles	Allele size range (bp)	No. of genotypes	Observed heterozygosity	Expected heterozygosity	F (null) (%)
Fh_2	TTGA	745	27	184–360	93	0.73	0.82	5.9
Fh_5	ACT	748	34	146–326	150	0.87	0.90	1.6
Fh_6	TAT	747	25	177–255	138	0.88	0.90	1.1
Fh_10	TAA	747	13	200–236	66	0.74	0.81	4.6
Fh_11	ATA	742	13	195–234	47	0.76	0.78	1.5
Fh_12	ATC	750	15	202–244	49	0.76	0.78	0.7
Fh_13	CAT	747	14	179–230	25	0.61	0.61	0.7
Fh_15	TATG	743	11	207–275	20	0.45	0.50	5.4
Mean	–	746.1	19.0	–	73.5	0.73	0.76	2.5

F (null): estimated null allele frequency

When analysing data from all flukes collectively, six loci (Fh_2, Fh_5, Fh_6, Fh_10, Fh_11 and Fh_15) deviated significantly from HWE as indicated by F_IS_ (for detailed F_IS_ values see Additional file 4: Supplementary Table 2). However, when deviations from HWE were assessed separately for each farm or host (Additional file 5: Supplementary Table 3), the number of loci deviating significantly from HWE decreased (mean number of deviating loci per farm/host = 0.6). Moreover, it was not always the same locus that showed deviations. There were only two farms with more than two deviating loci (farms J and O), suggesting that the observed deviations are farm-specific.

The assessment of LD for each pair of loci across all fluke samples revealed a significant positive correlation of allele frequencies for the pair Fh_2 and Fh_10 (*r*_*d*_ = 0.054, *P* = 0.028), while all other pairs of loci showed no significant LD (Additional file 4: Supplementary Table 2). At farm level, most farms showed no pairs of loci in LD (or at most only one pair), while four farms exhibited between 4 and 15 pairs of loci with significant LD (farms J, K, O and Q) (Additional file 5: Supplementary Table 3), again indicating a farm-specific bias rather than a general LD between pairs of loci.

The inbreeding coefficient F_IS_ was 0.0054 across all loci, resulting in an estimated rate of self-fertilisation of 1.1%. MLGs were analysed from the 730 flukes of which all eight loci were successfully amplified, and a total of 500 unique MLGs were identified across these samples (68.5%, 500/730). Out of the repeated MLGs (clones) (31.5%, 230/730), 80.9% (186/230) were found within the same host and 18.7% (43/230) were shared between hosts from the same farm. One MLG (0.4%, 1/230) was most likely shared between two farms, but unfortunately, information on the farm of origin was not available for one of these MLGs. The most prevalent MLG was identified 19 times in three different hosts, which were all from farm O. Table 5 presents a comparison of genetic diversity data between farms. Only samples with known farm of origin and successful amplification of all eight microsatellite loci were included (649 *F. hepatica* specimens from 58 livers).

**Table 5:** Farm-specific data on genetic diversity of German *F. hepatica* from multilocus genotypes (MLGs) derived from eight microsatellite loci

Farm	No. of livers	No. of flukes	Expected heterozygosity	No. of unique MLGs	Genotypic richness (*R*)
Farm A	2	12	0.70	10	0.82
Farm B	6	22	0.75	22	1.00
Farm C	1	6	0.74	6	1.00
Farm D	3	14	0.66	14	1.00
Farm E	1	4	0.46	3	0.67
Farm F	3	8	0.76	6	0.71
Farm G	1	11	0.70	9	0.80
Farm H	2	13	0.70	10	0.75
Farm I	2	17	0.74	17	1.00
Farm J	10	228	0.73	125	0.55
Farm K	9	62	0.76	47	0.75
Farm L	1	1	n.a.	1	n.a.
Farm M	2	4	0.68	4	1.00
Farm N	7	48	0.75	42	0.87
Farm O	3	107	0.66	40	0.37
Farm P	2	23	0.77	19	0.82
Farm Q	3	69	0.76	58	0.84
Mean	3.4	38.2	0.71	25.5	0.81

Only data from flukes with known farm of origin were included (649 liver flukes from 17 farms)

n.a.: not applicable

Genotypic richness (*R*), describing the proportion of unique genotypes in a farm, ranged from 0.37 to 1.00 in the sampled farms (mean: 0.81). Expected heterozygosity ranged from 0.46 to 0.77 per farm (mean: 0.71). After reducing MLGs to only one instance per host, the clone-corrected dataset included 554 MLGs. The AMOVA results showed that the farm explained 5.2% (*P* < 0.001, original dataset) or 4.6% (*P* < 0.001, clone-corrected dataset) of the genetic variation. The host accounted for 1.9% of the genetic variation (*P* < 0.001) using the original dataset, but had no significant influence when clones were excluded (0.4%, *P* = 0.281). The vast majority of genetic variation was found within hosts (original dataset: 92.9%, clone-corrected dataset: 95.0%) (Table 6). Overall, F_ST_ was 0.07 and 0.05 with and without prior clone correction, respectively, demonstrating that the subpopulations were highly similar. The mean frequency of private alleles per farm was 4.5%, and the resulting number of migrants of 3.5 indicated a high gene flow between farms [74].

**Table 6:** Analysis of molecular variance (AMOVA) of the multilocus genotypes (MLGs) from the microsatellite data of *F. hepatica* from Germany

Dataset	Source of variation	Sum of squares	Variance components	Percentage variation	*P*-value
Original dataset	Among farms	238.9	0.2	5.2%	< 0.001
	Among hosts within farms	165.8	0.1	1.9%	< 0.001
	Within hosts	3533.0	2.9	92.9%	< 0.001
	Total	3937.7	3.1	100%	n.a.
Clone-corrected dataset	Among farms	166.5	0.1	4.6%	< 0.001
	Among hosts within farms	126.8	0.0	0.4%	0.281
	Within hosts	2652.1	2.9	95.0%	< 0.001
	Total	2945.4	3.1	100%	n.a.

Samples were grouped by farm of origin and host. The AMOVA was performed using both the original dataset (*n* = 730 MLGs) and the clone-corrected dataset (*n* = 554 MLGs)

n.a.: not applicable

**Fig. 3:**
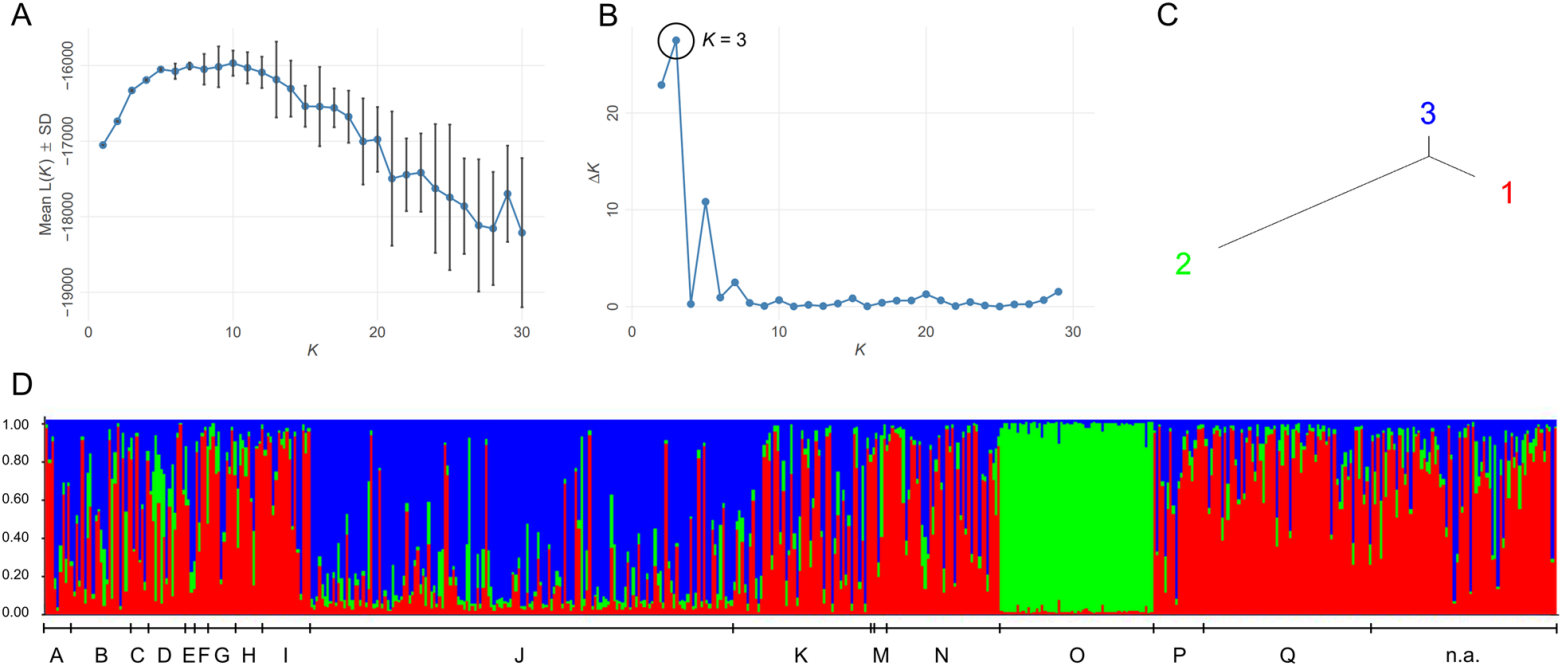
Population analysis results of the clone-corrected microsatellite dataset (*n* = 554 MLGs) of *F. hepatica* from Germany using the STRUCTURE software. The Evanno method [69] was applied for identifying the most likely number of populations (*K*) with a plot for mean L(*K*) (± standard deviation [SD]) over each run for each *K* value (**A**) and the Δ*K* plot (**B**). A tree plot for the most likely number of populations (*K* = 3) was created to visualize distances between the clusters (**C**). The bar plot shows the genetic heritage for each fluke (admixture heritage model) for *K* = 3 (**D**). The farms of origin are labelled, except for farm L, from which only one fluke was examined

**Fig. 4:**
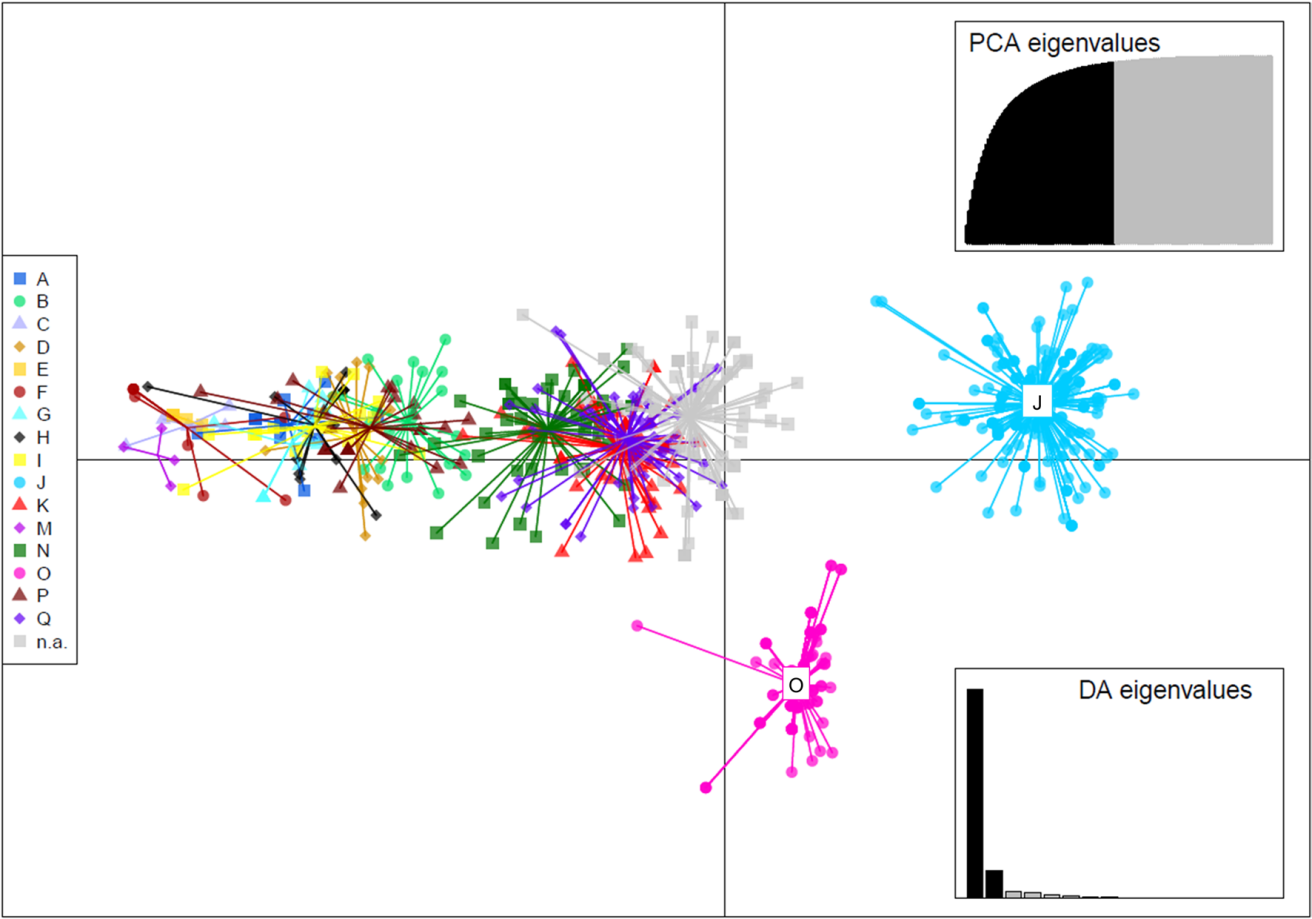
Discriminant analysis of principle components (DAPC) of the clone-corrected microsatellite dataset (*n* = 554 MLGs) of *F. hepatica* from Germany. Flukes are grouped by their farm of origin. Flukes for which information on the farm of origin was not available (n.a.) were grouped together. Samples were weighted by population size to mitigate biases caused by unequal sampling. One outlier originating from farm L was excluded

Bayesian clustering estimated a most likely number of populations of three, as indicated by the plot of mean likelihood L(*K*) (Fig. 3A) and the highest peak of Δ*K* (Fig. 3B), with the resulting populations one and three being more closely related to each other than to population two (Fig. 3C). Most flukes displayed an admixed heritage from all three populations, and in most cases, there was no obvious pattern by host or farm. However, on two farms, farm J and especially farm O, fluke heritage was dominated by a different population than on most of the other farms (Fig. 3D). The DAPC, in which the microsatellite data for each fluke were plotted according to their principal components, presented a similar picture (Fig. 4 and Additional file 6: Supplementary Fig. 3). Flukes from farms J and O differed the most from the others. Overall, flukes from the same farm often exhibited considerable distances between each other, indicating a high degree of intra-farm diversity.

### Phenotyping of German *F. hepatica* and associations between fluke morphometry and genotype

The measurements of all flukes revealed a median body length of 2.02 cm (95% CI 2.00–2.09 cm and range: 0.87–3.31 cm) and a median body width of 0.94 cm (95% CI 0.92–0.96 cm and range: 0.37–1.38 cm). The median ratio of body length to width was 2.20 (95% CI 2.17–2.24 and range: 0.96–4.91) and the median distance between the ventral sucker and the posterior end was 1.74 cm (95% CI 1.69–1.78 cm and range: 0.70–2.91 cm).

Spearman’s rank correlation showed a slight negative correlation between the number of flukes in the liver and fluke length (*ρ* = −0.12, *P* = 0.003), as well as fluke width (ρ = −0.15, *P* < 0.001). Without correcting for other influences, fluke sizes varied between the two mitochondrial clusters (Fig. 1). Flukes belonging to cluster 2 were longer than flukes belonging to cluster 1 (median length of 2.00 cm in cluster 1 versus median length of 2.15 cm in cluster 2). Moreover, flukes from cluster 2 were wider than flukes from cluster 1 (median width of 0.93 cm in cluster 1 versus median width of 0.97 cm in cluster 2).

**Fig. 5:**
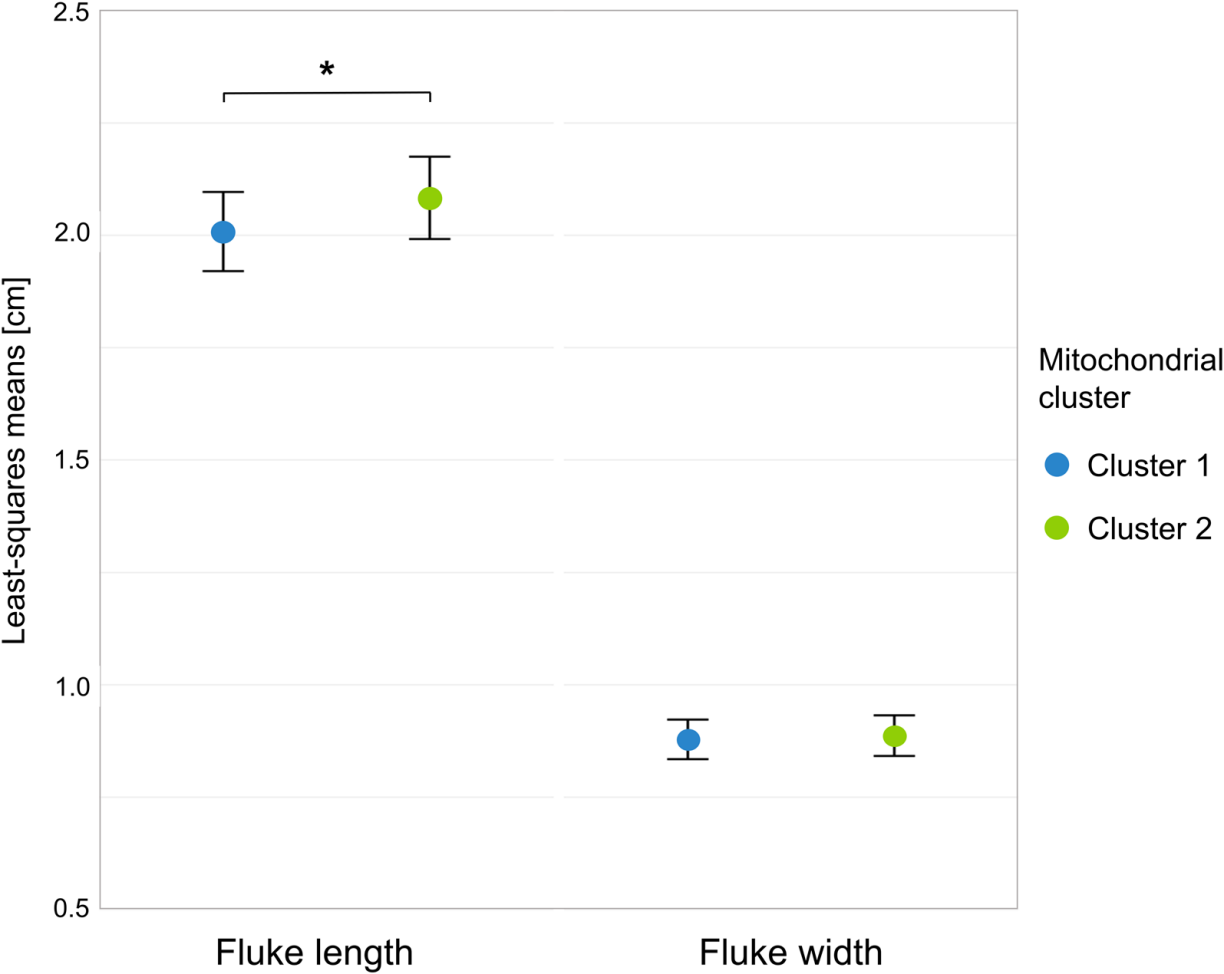
Least-squares means for length and width of German *F. hepatica* in each mitochondrial cluster, adjusted for all variables included in the linear mixed models. The error bars represent standard errors, and the asterisk indicates statistical significance in the pairwise comparison (*P = 0.030)

Results of the linear mixed model corrected for the effects of fluke count, sampling month and host showed that mitochondrial cluster (chi-squared test, *χ*^2^ = 4.73, degrees of freedom [df] = 1 and *P* = 0.029) and farm (chi-squared test, *χ*^2^ = 93.32, df = 15 and *P* = 0.015) significantly influenced fluke length. Flukes belonging to cluster 2 were significantly longer than flukes belonging to cluster 1 (least-squares means of 2.01 cm in cluster 1 and 2.08 cm in cluster 2, *P* = 0.030) (Fig. 5). Although the farm had a significant effect on fluke length, pairwise comparisons of least-squares means between farms revealed no statistically significant differences (Additional file 7). Fluke width was not significantly affected by the mitochondrial cluster (chi-squared test, *χ*^2^ = 0.437, df = 1 and *P* = 0.509). However, the farm had a significant effect on fluke width (chi-squared test, *χ*^2^ = 35.50, df = 15 and *P* = 0.002). Pairwise comparisons of least-squares means for fluke width were not statistically significant, neither between mitochondrial clusters (0.88 cm in cluster 1 and 0.89 cm in cluster 2, *P* = 0.510) (Fig. 5) nor between farms (Additional file 7).

## Discussion

Knowledge of population genetics of a parasite can provide valuable insights into its distribution and evolutionary potential. This study presents the first report on genetic diversity and population structure of *F. hepatica* in Germany, analysing nuclear (ITS-1) and mitochondrial (*cox1* and *nad1*) sequences as well as eight nuclear microsatellite markers.

While the two mitochondrial genes *cox1* and *nad1* showed considerable differences between flukes, the low variation found in the ITS-1 region was not surprising, as nuclear sequences rarely exhibit substantial intraspecific variation [19]. However, ITS-1 is used as a marker to distinguish between species such as *F. hepatica* and *Fasciola gigantica* [20, 43, 75] and allowed unambiguous species classification of all examined flukes as *F. hepatica*. The two mitochondrial genes *cox1* and *nad1* both showed a three to four times higher percentage of variable sites than that reported from Eastern Europe, Western Asia [26], Iran [23], China [27] and Australia [76], where mtDNA was sequenced from 119, 208, 90 and 144 liver flukes, respectively. The percentage of non-synonymous substitutions was approximately eight times higher for the combined *cox1*/*nad1* sequences than for those from Eastern Europe and Western Asia, which had only one non-synonymous single nucleotide polymorphism [26]. Non-synonymous substitutions are considered particularly relevant because they lead to changes in the amino acid sequence and therefore may affect the function of the translated protein. Both proteins (cytochrome c oxidase subunit 1 and NADH dehydrogenase subunit 1) are involved in processes of oxidative phosphorylation, and may therefore impact the parasite’s metabolic efficiency and ability to adapt to different environmental conditions [77]. Accordingly, a link between nucleotide substitutions in *cox1* and *nad1* genes and drug resistance has often been hypothesised [21, 76]. The diversity markers *π* and Hd were found to be lower than in Eastern European, Western Asian and Chinese *F. hepatica* [26, 27], but higher than in Spanish, Algerian and Iranian flukes [20, 21, 23], indicating geographical differences. However, discrepancies in sampling design, methodology, and possible genotyping errors may impede the comparability between studies. Additionally, the number of flukes examined may be a contributing factor, as most studies involved a much smaller sample size than the present study. The analysed flukes showed an overall high degree of genetic diversity, which is associated with enhanced adaptability, e.g. to new definitive or intermediate host species, climatic changes or anthelmintic treatment [27, 78]. Interestingly, there were considerable discrepancies in *π* and Hd between farms, demonstrating the existence of farm-specific dynamics. A decrease in diversity, as seen in some farms, can occur after a bottleneck event, which could be caused by treatment, extreme climatic events significantly reducing fluke stages on pasture or a founder effect after introduction into a previously uninfected herd [36]. Neutrality tests, which assess whether the sampled population deviates from a neutral evolutionary model by analysing the frequency pattern of polymorphisms, were all significantly negative. Negative neutrality tests imply an excess of low-frequency polymorphisms, which are indicative of rare alleles [48, 79]. This violation of neutrality could be caused by a selective advantage of an allele, a previous negative selection or a recent population expansion [48].

Phylogenetic analysis of the *cox1* and *nad1* sequences revealed two main clusters. The percentage of nucleotide variations between clusters can be used to estimate how long ago the clusters separated (mitochondrial clock). If a mitochondrial clock of 2–4% divergence per million years is applied [26, 80], the two clusters found in this study would have separated approximately 200,000 (4% divergence) to 400,000 years (2% divergence) ago. Interestingly, previous studies also have identified two mitochondrial clusters, which diverged 250,000 years ago on the basis of a 4% mitochondrial clock in *F. hepatica* from the Netherlands [80] and 300,000 years ago on the basis of a 2% mitochondrial clock in *F. hepatica* from Eastern Europe and Western Asia [26]. Unfortunately, the analysis of different genes or lack of access to the sequences from these studies does not allow a conclusion as to whether these clusters have the same origin as the two clusters found in the present study. The haplotype network shows that haplotypes within the two clusters tend to differ by only a few nucleotides. This is typical for relatively recent mutations, which could indicate a recent population expansion [80]. The two clusters revealed little association with the farm or host of origin, as indicated by the overall low F_ST_ as a measure of the degree of population structuring. This matches reports of weak farm- or region-associated population structure based on mitochondrial gene sequencing in the Netherlands [80], Eastern Europe, Western Asia [26], China [27], Algeria [20] and Argentina [28]. However, an association between polymorphisms in the mtDNA and the sampling region was found in Brazilian *F. hepatica*, which was hypothesised to be caused by large geographical distances combined with little cattle trade [25]. While cattle trade is presumed to be the most important factor for the circulation of haplotypes between farms [81], other possible paths of transmission include wild ruminants acting as carrier hosts [82], or even the transfer of the intermediate snail host by humans or wildlife, e.g. birds [83]. A high level of haplotype circulation between farms, i.e. a high gene flow, as observed in the present study, can promote the spread of new alleles, including drug resistance genes [35]. The clustering of the identified haplotypes in this study with sequences from various other countries in a global phylogenetic tree suggests that this high gene flow likely extends across national borders.

Microsatellite analysis showed that all eight loci were highly polymorphic. Allele and genotype frequencies were similar to those previously described in the UK after genotyping 1579 *F. hepatica* from sheep and cattle [14] and in France, where 1148 liver flukes from different host species were analysed [84]. Null allele frequencies were estimated to be < 6% for all loci, demonstrating an adequate amplification of all loci, and therefore excluding an interference with population genetic analyses [85]. Other factors that potentially affect genetic analyses, such as deviations from HWE and LD between loci, were also investigated. Across the entire dataset, six loci showed significant deviations from HWE by exhibiting heterozygote deficiencies. There are numerous potential causes for deviations from HWE including selection, mutation, population structure, overlapping generations, limited population size, inbreeding or genotyping error [64]. To gain further insight, deviations from HWE were calculated at farm level, revealing minimal deviations except on two farms. This suggests that the observed deviations are likely to be farm-specific anomalies rather than affecting the whole sampled *F. hepatica* population or being caused by a genotyping error. Similar farm-specific deviations were found for LD between pairs of loci, where again the same two farms showed the most pairs of loci with a positive correlation of allele frequencies.

The low rate of self-fertilisation calculated from F_IS_ values across all loci confirms that *F. hepatica* prefers to cross-fertilize, which is beneficial for maintaining genetic diversity, as it promotes heterozygosity [86]. In line with this finding, mean expected heterozygosity was high for most loci and similar to heterozygosity levels in British *F. hepatica* reported by Beesley et al. [14]. High heterozygosity is associated with evolutionary advantages; Zintl et al. [87] found evidence that heterozygous *F. hepatica* may have an advantage over homozygous *F. hepatica* with respect to establishment in the final host. While the proportion of clones may affect genetic diversity as a consequence of inbreeding [88, 89], no association between heterozygosity levels and clone frequencies, i.e. genotypic richness, was observed in this study. In line with this finding, Prugnolle et al. [90] proposed that heterozygosity is not affected by clones as long as they still mate randomly.

Overall, the degree of structuring by farm or host was low in the present study. Consistent with this lack of genetic differentiation between subpopulations, the high number of migrants suggested a considerable gene flow between farms [74]. Population structure as indicated by F_ST_ was similarly low to that observed in 587 *F. hepatica* from ten locations in Spain [35] and pooled *F. hepatica* from ten locations in Cuba [37]; however, it was slightly higher than that reported in the UK, where the sampled *F. hepatica* population (*n* = 1579 flukes) has been described as panmictic [14]. The main reason for the complete lack of structure in the UK was hypothesised to be the large volume of cattle trade [14]. While the influence of the farm on genetic variation was small but significant in the present study, the host only had an influence when clones were included in the dataset. Accordingly, most clones were found in the same host. Vilas et al. [35], who also observed an accumulation of *F. hepatica* clones within the same host in sheep and cattle, explained this with the clumped transmission of metacercariae. Snails, in which clonal amplification occurs, have been shown to shed multiple cercariae at once [91], which, combined with the low mobility of metacercariae, leads to their accumulation in small areas of the pasture and subsequent ingestion by the same host while grazing. Other factors, such as a genetic adaptation of some flukes to certain hosts might also have an influence on the distribution of parasite genotypes.

Multivariate and Bayesian population structure analyses revealed that two farms were genetically distinct from the others. Interestingly, these two farms also deviated the furthest from HWE, showed most pairs of loci in LD and had the highest proportion of clones, i.e. the lowest genotypic richness. These findings suggest that those two farms are isolated from the others [92], as isolated populations are typically genetically distinct from the rest of the population and tend to be less diverse [93]. A geographical isolation can be ruled out for at least one of the farms, while the exact location of the other farm was not available. It is a possibility that these farms are engaging in minimal or no cattle trade, which prevents the introduction of new genetic material. Accordingly, the *F. hepatica* population on a farm in Argentina with no reported cattle trade was found to be genetically distinct from other farms [15]. However, no information on the cattle trade practices employed on the farms in question were available.

Both the mtDNA and the microsatellite datasets indicate a high potential for adaptability and a rapid spread of new alleles, which could facilitate the quick development and spread of drug resistance and should be considered in resistance monitoring. While both datasets indicate a low degree of structuring by host or farm, the detected population structure differed, showing two clearly separated clusters on the basis of mtDNA sequencing and two genetically distinct farms on the basis of microsatellite analysis. A discrepancy in population structure detected by mitochondrial sequences and nuclear microsatellite markers has also been documented in other organisms and is attributed to disparate mutation rates and ways of inheritance on mitochondrial and nuclear levels [32, 38, 39]. The haploid mtDNA is uniparentally inherited without recombination, has a small effective population size and is therefore strongly affected by genetic drift, which can result in pronounced population subdivision [31]. In contrast, nuclear alleles originate from both parents and are recombined in each new generation, which leads to more genetic variation compared with mtDNA. This renders nuclear markers particularly powerful for the differentiation of closely related individuals. However, the high variation between individuals may obscure differences between groups [31]. Thus, the two clusters identified through mtDNA sequencing in the present study may not be detectable in nuclear markers. This highlights the benefit of a comparative analysis of mitochondrial and nuclear DNA, so that structural patterns are not overlooked.

The liver flukes measured in the present study had a similar mean body length compared with flukes collected from cattle livers in France, but were longer than flukes collected from cattle in Mexico, Bolivia and Spain [94]. The measured body width was smaller than that of flukes from France and Spain, but wider than that from Mexico and Bolivia [94]. There was a slight negative correlation between fluke size and the number of flukes in the liver, which is in line with reports of a decreasing fluke size when more flukes were present in rat livers [95]. Given that genetic markers have the potential to alter phenotypic characteristics, a comparison between fluke sizes and the genotyping results was conducted. Even microsatellites located in non-coding regions have been shown to alter gene expression in humans [96]. Furthermore, a correlation between microsatellite data and dsRNA sequences has been observed for *Cryptosporidium* spp. [97], indicating a possible link with phenotypic traits [87]. Significant differences in fluke length were detected between the two mitochondrial clusters in linear mixed model analysis, with flukes from cluster 2 being 3.5% longer than those from cluster 1 on average. The effect of the microsatellite markers could not be assessed directly owing to the high abundance of unique genotypes. Potential influences of fluke genotypes are included in the factor ‘farm’, but genotypes might also affect the fluke count, as the ability of a fluke to successfully infect the final host has been suggested to be influenced by fluke genetics [87]. The lack of statistically significant differences in pairwise comparisons of fluke sizes between farms can largely be attributed to the small sample sizes in several farms. Furthermore, other factors that may impact fluke size have not been considered, such as the age of the flukes [98]. Considering that naturally infected animals were investigated, the date of infection, and therefore, the age of the flukes was unknown. However, since the majority of hosts harboured flukes from both mitochondrial clusters, it can be assumed that fluke age was similarly distributed across both clusters.

Further research could provide a deeper understanding of the factors influencing or being influenced by fluke genetics, such as farm management and cattle trade practices, or the inclusion of longitudinal or drug resistance data. Furthermore, larger and more evenly distributed sample sizes per farm would be beneficial. As sample sizes between farms varied strongly in this study, farms with lower sample sizes might be underrepresented. Even when correcting for sample size, as was done prior to the DAPC in the present study, a potential bias due to unequal sampling cannot be ruled out.

## Conclusions

*Fasciola hepatica* in German dairy cows showed high genetic diversity as evidenced by both mtDNA and microsatellite markers. There was little structuring by host or farm, indicating a high level of gene flow between farms. The detected population structure differed between mitochondrial and nuclear markers. On the basis of the mitochondrial *cox1* and *nad1* sequences, two genetic clusters with little host- or farm-specific association could be distinguished, whereas the analysis of eight nuclear microsatellite markers revealed two farms that were genetically distinct. The results highlight the advantage of studying genetic markers of different origins, as this may reveal different existing population structures. This is the first study analysing the effect of *F. hepatica* genotyping data on fluke size, revealing significant differences in fluke length between mitochondrial clusters.

## Supplementary Information

Supplementary file 1: Figure 1. Map of northwestern and central Germany showing the 14 dairy farms with known locations (farms A to N) from which *F. hepatica* were sampled from slaughtered cows. German federal states are abbreviated as follows: SH = Schleswig-Holstein, LS = Lower Saxony, HE = Hesse.

Supplementary file 2: Figure 2. Maximum-likelihood consensus trees comparing the two most frequent mitochondrial haplotypes (H2 and H22) of German *F. hepatica* with global *cox1* (A) and *nad1* (B) reference sequences retrieved from NCBI GenBank. Evolutionary model: Hashinawa-Kishono-Yano+F, bootstrap values >50 are shown.

Supplementary file 3: Table 1. Allele lengths of eight microsatellite loci (Fh_2, Fh_5, Fh_6, Fh_10, Fh_11, Fh_12, Fh_13 and Fh_15) of German *F. hepatica*. Sample ID consists of liver (L) and fluke (F) number and information on the farm of origin is provided (n.a.=not applicable, no information on the farm of origin).

Supplementary file 4: Table 2. Deviations from Hardy-Weinberg Equilibrium (HWE) for each microsatellite locus and assessment of linkage disequilibrium (LD) for each pair of loci in *F. hepatica* from Germany. Deviations from HWE were assessed by calculating the inbreeding coefficient F_IS_ [59]. To test for LD, the index of association (*I*_*A*_) [60] and the standardised index of association (*r*_*d*_) [61] were used and *P*-values were Bonferroni corrected. Statistically significant values (*P*≤0.05) are marked with asterisks.

Supplementary file 5: Table 3. Deviations from Hardy-Weinberg Equilibrium (HWE) in *F. hepatica* from Germany, based on F_IS_ values that differ significantly (*P*≤0.05) from 0 in either direction, and pairs of loci in linkage disequilibrium (LD), based on a significant standardized index of association (*r*_*d*_) (*P*≤0.05 after Bonferroni correction), assessed for each farm or host, if data on farm of origin was not available. The two farms with the highest numbers are marked with asterisks.

Supplementary file 6: Figure 3. Results of the Discriminant Analysis of Principle Components (DAPC) of the clone-corrected microsatellite dataset (n=554 MLGs) of *F. hepatica* from Germany, shown in a three-dimensional plot. Flukes were grouped by their farm of origin (n.a.=not applicable, no information on the farm of origin). Samples were weighted by population size to mitigate biases caused by unequal sampling. One outlier originating from farm L was excluded.

Supplementary file 7: Figure 4. Least-squares means for German *F. hepatica* length and width in each farm, adjusted for all variables included in the linear mixed models. The error bars represent standard errors. Pairwise comparisons did not result in statistically significant differences between farms. Farm L was excluded from this analysis because only one fluke was collected from that farm. All flukes with unknown farm of origin were grouped together (n.a.=not applicable, no information on the farm of origin).

## Data Availability

Data supporting the findings of this study are available within the article and its additional files. Generated sequences were deposited in GenBank under accession numbers PQ742167-PQ742169 (ITS-1), PQ742177-PQ742950 (*cox1*) and PQ759109-PQ759882 (*nad1*).

## Abbreviations

mtDNA: Mitochondrial DNA
Hd: Haplotype diversity
ITS: Internal transcribed spacer
*cox1*: Cytochrome C oxidase gene subunit 1
*nad1*: Nicotinamide dehydrogenase gene subunit 1
AMOVA: Analysis of molecular variance
MLG: Multilocus genotype
HWE: Hardy–Weinberg equilibrium
LD: Linkage disequilibrium
DAPC: Discriminant analysis of principal components
