# Supplementary material for: Population genetic analysis of the liver fluke Fasciola hepatica in German dairy cattle reveals high genetic diversity and associations with fluke size
Source: Parasit Vectors. 2025 Feb 13;18:51. doi: 10.1186/s13071-025-06701-6 (PMC11827327; doi:10.1186/s13071-025-06701-6)

**Additional file 1: Figure S1**. Map of northwestern and central Germany showing the 14 dairy farms with known locations (farms A to N) from which *F. hepatica* were sampled from slaughtered cows. German federal states are abbreviated as follows: SH = Schleswig-Holstein, LS = Lower Saxony, HE = Hesse.


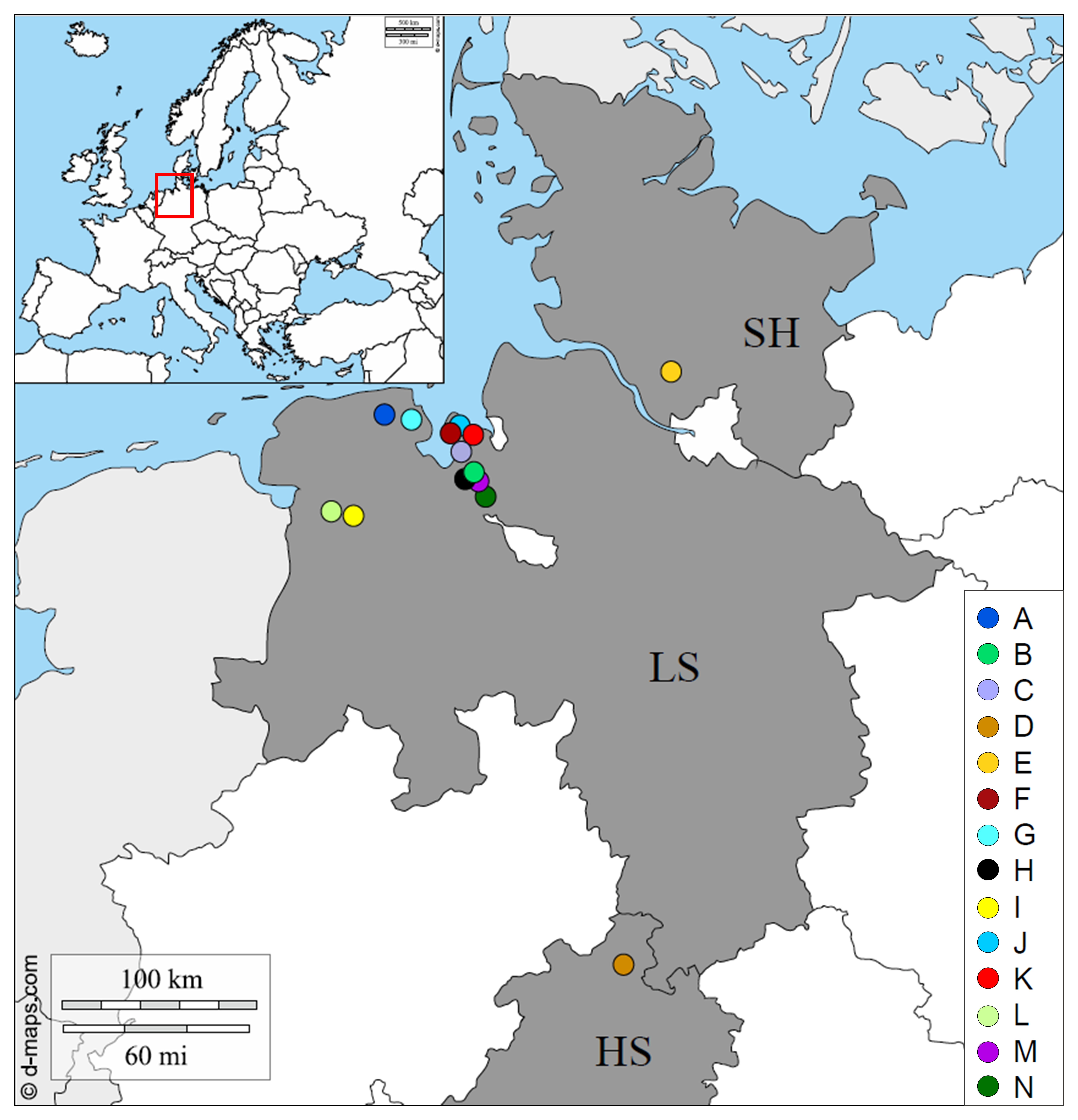

Supplement: Supplementary file 1 — Figure 1. Map of northwestern and central Germany showing the 14 dairy farms with known locations (farms A to N) from which F. hepatica were sampled from slaughtered cows. German federal states are abbreviated as follows: SH = Schleswig-Holstein, LS = Lower Saxony, HE = Hesse. [file 13071_2025_6701_MOESM1_ESM.docx]
