# Supplementary material for: Population genetic analysis of the liver fluke Fasciola hepatica in German dairy cattle reveals high genetic diversity and associations with fluke size
Source: Parasit Vectors. 2025 Feb 13;18:51. doi: 10.1186/s13071-025-06701-6 (PMC11827327; doi:10.1186/s13071-025-06701-6)

**Additional file 2: Figure S2**. Maximum-likelihood consensus trees comparing the two most frequent mitochondrial haplotypes (H2 and H22) of German *F. hepatica* with global *cox1* (A) and *nad1* (B) reference sequences retrieved from NCBI GenBank. Evolutionary model: Hashinawa-Kishono-Yano+F, bootstrap values >50 are shown.


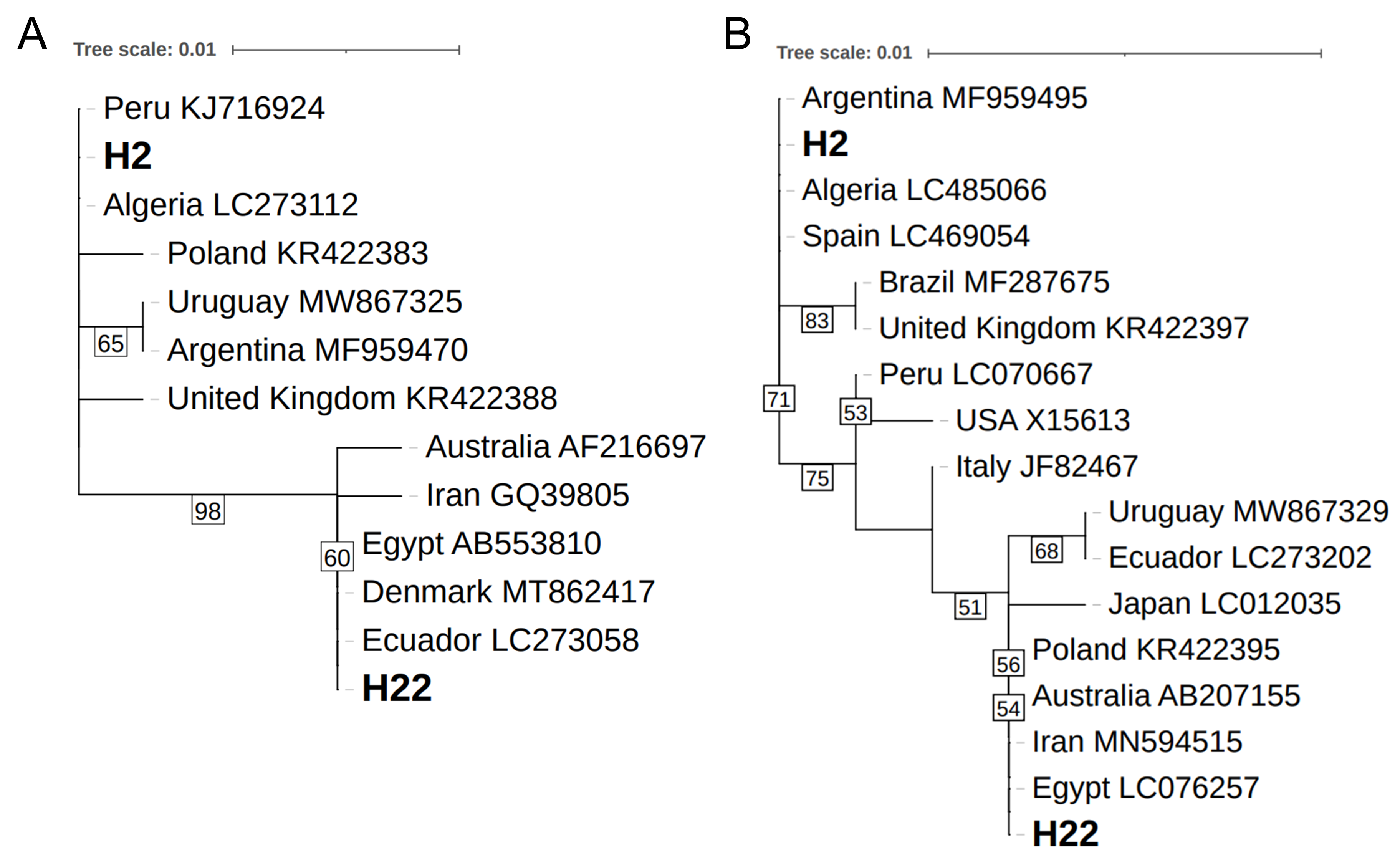

Supplement: Supplementary file 2 — Figure 2. Maximum-likelihood consensus trees comparing the two most frequent mitochondrial haplotypes (H2 and H22) of German F. hepatica with global cox1 (A) and nad1 (B) reference sequences retrieved from NCBI GenBank. Evolutionary model: Hashinawa-Kishono-Yano+F, bootstrap values >50 are shown. [file 13071_2025_6701_MOESM2_ESM.docx]
