# Supplementary material for: Population genetic analysis of the liver fluke Fasciola hepatica in German dairy cattle reveals high genetic diversity and associations with fluke size
Source: Parasit Vectors. 2025 Feb 13;18:51. doi: 10.1186/s13071-025-06701-6 (PMC11827327; doi:10.1186/s13071-025-06701-6)
Supplement: Supplementary file 4 — Table 2. Deviations from Hardy-Weinberg Equilibrium (HWE) for each microsatellite locus and assessment of linkage disequilibrium (LD) for each pair of loci in F. hepatica from Germany. Deviations from HWE were assessed by calculating the inbreeding coefficient FIS [59]. To test for LD, the index of association (IA) [60] and the standardised index of association (rd) [61] were used and P-values were Bonferroni corrected. Statistically significant values (P≤0.05) are marked with asterisks. [file 13071_2025_6701_MOESM4_ESM.docx]

**Additional file 4: Table S2.** Deviations from Hardy-Weinberg Equilibrium (HWE) for each microsatellite locus and assessment of linkage disequilibrium (LD) for each pair of loci in *F. hepatica* from Germany. Deviations from HWE were assessed by calculating the inbreeding coefficient F_IS_ [59]. To test for LD, the index of association (*I_A_*) [60] and the standardised index of association (*r_d_*) [61] were used and *P*-values were Bonferroni corrected. Statistically significant values (*P*<0.05) are marked with asterisks.

| **Deviations from HWE** | | | **LD** | | | | |
| --- | --- | --- | --- | --- | --- | --- | --- |
| Locus | F_IS_ | *P*-value | Pair of loci | *I_A_* | *P*-value | *r_d_* | *P*-value |
| Fh_2 | 0.109 | <0.001* | Fh_2:Fh_5 | -0.006 | 1.000 | -0.006 | 1.000 |
| Fh_5 | 0.050 | <0.001* | Fh_2:Fh_6 | 0.012 | 1.000 | 0.012 | 1.000 |
| Fh_6 | 0.030 | 0.009* | Fh_2:Fh_10 | 0.055 | 0.028* | 0.054 | 0.028* |
| Fh_10 | 0.076 | <0.001* | Fh_2:Fh_11 | 0.013 | 1.000 | 0.013 | 1.000 |
| Fh_11 | 0.025 | 0.003* | Fh_2:Fh_12 | 0.036 | 0.084 | 0.036 | 0.084 |
| Fh_12 | -0.007 | 0.372 | Fh_2:Fh_13 | -0.001 | 1.000 | -0.001 | 1.000 |
| Fh_13 | 0.017 | 0.310 | Fh_2:Fh_15 | 0.016 | 1.000 | 0.017 | 1.000 |
| Fh_15 | 0.137 | 0.010* | Fh_5:Fh_6 | 0.024 | 0.056 | 0.024 | 0.056 |
|  |  |  | Fh_5:Fh_10 | 0.020 | 1.000 | 0.021 | 1.000 |
|  |  |  | Fh_5:Fh_11 | -0.001 | 1.000 | -0.001 | 1.000 |
|  |  |  | Fh_5:Fh_12 | 0.029 | 0.196 | 0.029 | 0.196 |
|  |  |  | Fh_5:Fh_13 | -0.003 | 1.000 | -0.003 | 1.000 |
|  |  |  | Fh_5:Fh_15 | -0.022 | 1.000 | -0.023 | 1.000 |
|  |  |  | Fh_6:Fh_10 | 0.023 | 0.448 | 0.023 | 0.448 |
|  |  |  | Fh_6:Fh_11 | -0.005 | 1.000 | -0.006 | 1.000 |
|  |  |  | Fh_6:Fh_12 | -0.001 | 1.000 | -0.001 | 1.000 |
|  |  |  | Fh_6:Fh_13 | 0.023 | 0.196 | 0.024 | 0.196 |
|  |  |  | Fh_6:Fh_15 | -0.018 | 1.000 | -0.019 | 1.000 |
|  |  |  | Fh_10:Fh_11 | 0.021 | 1.000 | 0.021 | 1.000 |
|  |  |  | Fh_10:Fh_12 | 0.030 | 0.280 | 0.030 | 0.280 |
|  |  |  | Fh_10:Fh_13 | -0.004 | 1.000 | -0.004 | 1.000 |
|  |  |  | Fh_10:Fh_15 | -0.030 | 1.000 | -0.030 | 1.000 |
|  |  |  | Fh_11:Fh_12 | 0.017 | 1.000 | 0.017 | 1.000 |
|  |  |  | Fh_11:Fh_13 | 0.014 | 1.000 | 0.014 | 1.000 |
|  |  |  | Fh_11:Fh_15 | 0.024 | 1.000 | 0.024 | 1.000 |
|  |  |  | Fh_12:Fh_13 | 0.014 | 1.000 | 0.014 | 1.000 |
|  |  |  | Fh_12:Fh_15 | 0.011 | 1.000 | 0.011 | 1.000 |
|  |  |  | Fh_13:Fh_15 | 0.000 | 1.000 | 0.000 | 1.000 |
