# Supplementary material for: Population genetic analysis of the liver fluke Fasciola hepatica in German dairy cattle reveals high genetic diversity and associations with fluke size
Source: Parasit Vectors. 2025 Feb 13;18:51. doi: 10.1186/s13071-025-06701-6 (PMC11827327; doi:10.1186/s13071-025-06701-6)
Supplement: Supplementary file 5 — Table 3. Deviations from Hardy-Weinberg Equilibrium (HWE) in F. hepatica from Germany, based on FIS values that differ significantly (P≤0.05) from 0 in either direction, and pairs of loci in linkage disequilibrium (LD), based on a significant standardized index of association (rd) (P≤0.05 after Bonferroni correction), assessed for each farm or host, if data on farm of origin was not available. The two farms with the highest numbers are marked with asterisks. [file 13071_2025_6701_MOESM5_ESM.docx]

**Additional file 5: Table S3.** Deviations from Hardy-Weinberg Equilibrium (HWE) in *F. hepatica* from Germany, based on F_IS_ values that differ significantly (*P*<0.05) from 0 in either direction, and pairs of loci in linkage disequilibrium (LD), based on a significant standardized index of association (r*_d_*) (*P*<0.05 after Bonferroni correction), assessed for each farm or host, if data on farm of origin was not available. The two farms with the highest numbers are marked with asterisks.

|  | **Farm or host^a^** | **No. of loci with significant deviations from HWE (names of the deviating loci)** | **No. of pairs of loci in significant LD** |
| --- | --- | --- | --- |
| Farm | A | 0 | 1 |
|  | B | 2 (Fh_2, Fh_10) | 0 |
|  | C | 0 | 0 |
|  | D | 1 (Fh_5) | 0 |
|  | E | 0 | 0 |
|  | F | 0 | 0 |
|  | G | 0 | 1 |
|  | H | 0 | 1 |
|  | I | 1 (Fh_6) | 0 |
|  | J* | 3* (Fh_2, Fh_10, Fh_11) | 12* |
|  | K | 1 (Fh_5) | 9 |
|  | L | n.d. | 0 |
|  | M | 0 | 0 |
|  | N | 0 | 0 |
|  | O* | 3* (Fh_10, Fh_11, Fh_13) | 15* |
|  | P | 1 (Fh_2) | 0 |
|  | Q | 1 (Fh_13) | 4 |
| Host | x1 | 0 | 0 |
|  | x2 | 0 | 0 |
|  | x3 | 0 | 0 |
|  | x4 | n.d. | 0 |
|  | x5 | 0 | 0 |
|  | x6 | 1 (Fh_11) | 0 |
|  | x7 | n.d. | 0 |
|  | x8 | n.d. | 0 |
|  | x9 | 1 (Fh_13) | 0 |
|  | x10 | 0 | 0 |
|  | x11 | 0 | 0 |
|  | x12 | 0 | 0 |
|  | x13 | 1 (Fh_10) | 0 |
| Mean |  | 0.62 | 1.43 |

n.d. = not determined due to small sample size

^a^ Farms are labelled from A to Q, hosts with unknown farm of origin are labelled from x1 to x13
