# Supplementary material for: Population genetic analysis of the liver fluke Fasciola hepatica in German dairy cattle reveals high genetic diversity and associations with fluke size
Source: Parasit Vectors. 2025 Feb 13;18:51. doi: 10.1186/s13071-025-06701-6 (PMC11827327; doi:10.1186/s13071-025-06701-6)

**Additional file 7: Figure S4**. Least-squares means for German *F. hepatica* length and width in each farm, adjusted for all variables included in the linear mixed models. The error bars represent standard errors. Pairwise comparisons did not result in statistically significant differences between farms. Farm L was excluded from this analysis because only one fluke was collected from that farm. All flukes with unknown farm of origin were grouped together (n.a.=not applicable, no information on the farm of origin).


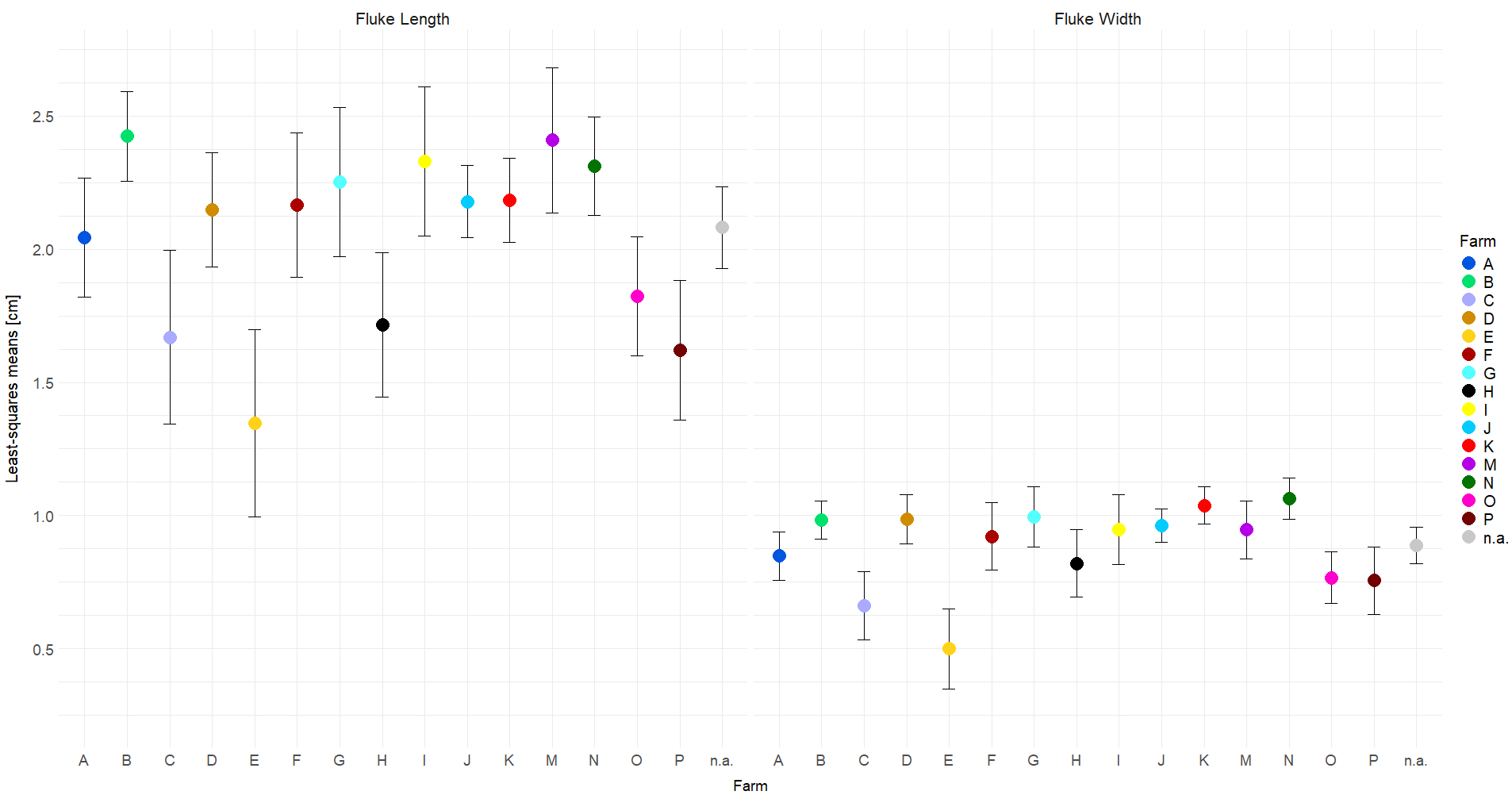

Supplement: Supplementary file 7 — Figure 4. Least-squares means for German F. hepatica length and width in each farm, adjusted for all variables included in the linear mixed models. The error bars represent standard errors. Pairwise comparisons did not result in statistically significant differences between farms. Farm L was excluded from this analysis because only one fluke was collected from that farm. All flukes with unknown farm of origin were grouped together (n.a.=not applicable, no information on the farm of origin). [file 13071_2025_6701_MOESM7_ESM.docx]
